# Editorial: Mast Cells in Itch, Pain and Neuro-Inflammation

**DOI:** 10.3389/fncel.2019.00521

**Published:** 2019-12-03

**Authors:** Rashid Giniatullin, Kalpna Gupta, Theoharis Theoharides

**Affiliations:** ^1^AIV Institute, University of Eastern Finland Kuopio, Kuopio, Finland; ^2^Laboratory of Neurobiology, Kazan Federal University, Kazan, Russia; ^3^Hematology/Oncology Department of Medicine, University of California, Irvine, Irvine, CA, United States; ^4^Department of Immunology, Tufts University School of Medicine, Boston, MA, United States

**Keywords:** mast cells, pain, itch, neuroinflammation, neuro-immune synapse

The present e-book contains a collection of articles on the emerging role of mast cells in various immunological, neurological, and cerebrovascular diseases. The growing interest to this cell type is based on the accumulating findings that mast cells are the key players in the so called “*neuro-immune synapse*” underlying pathologic processes both in the periphery and the central nervous system (CNS). This concept suggests that in addition to providing immune surveillance and triggering the inflammatory response, mast cells can also provide a fast-bidirectional crosstalk with neurons (Figure 1).

**Figure 1:**
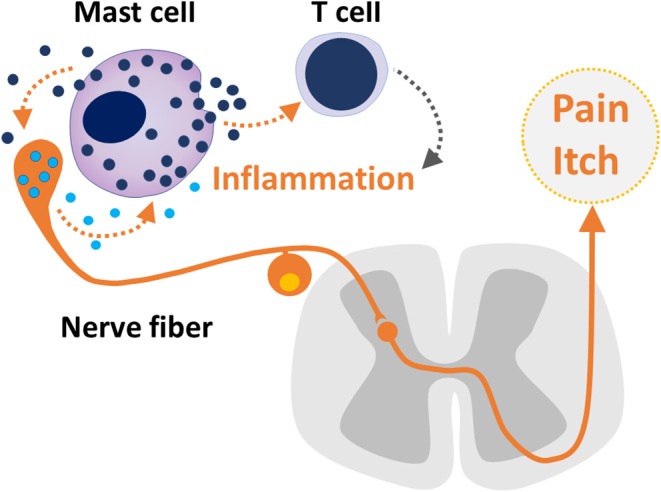
The bidirectional crosstalk between sensory neuron and mast cells, mast cell - T cell communication in inflammation and nociceptive signaling underlaying pain and itch.

It has been known for a long time that mast cells are ubiquitously present in many tissues, located close or even in direct contact with blood vessels and nerve fibers. The tissues where mast cells are most abundant are body surfaces exposed to the environment, such as skin, lungs, and gut. However, mast cells have a significant presence in the meninges and certain brain regions, especially the diencephalon, under normal and/or pathological conditions. The latter fact is of special interest, as these brain mast cells could be increased or stimulated in certain neurological diseases. Mast cells have been increasingly reported as important contributors to pain conditions such as fibromyalgia, headache, itch, sickle cell disease and brain disorders, stroke, traumatic brain injury, and various neurodegenerative disorders. However, mast cells display tremendous heterogeneity in their location, structure, content of active molecules, responsiveness to the surrounding environment and function. Hence, the mast cell phenotype cannot be generalized and varies in different pathophysiological conditions, including nociception and neurodegenerative disorders. Mast cells may also have a disease modifying effects including the pathobiology of pain, the time-course and the outcome of neurodegenerative diseases such as Alzheimer's and Parkinson disease as well as stroke and traumatic brain injury.

This premise requires establishing the disease-specific role of mast cells. Many of these interesting novel lines of research are presented in the current collection.

The present e-book consists of 17 articles including one brief research report, one hypothesis and theory article, seven original research articles, five reviews, and three mini-reviews.

Although the idea of the *neuro-immune synaps*e is presented in direct or indirect manner in most of the papers, some of them are specifically devoted to this concept. Thus, Mittal et al. present the concept of *neuro-immune synapse* and the critical role of mast cells in neuroinflammation. In particular, they discuss the novel aspect of mast cell interaction with the nervous system through extracellular vesicles, tunneling nanotubes, and extracellular traps that may contribute to various pathological brain conditions.

The paper by Magadmi et al. is devoted to the organization of the neuro-immune synapse and suggests the contribution of the CADM1-dependent mechanism for adhesion of the key partners involved in the *neuro-immune synapse*.

Siiskonen and Harvima present the concept of the *neuro-immune synapse* for skin disorders. They reviewed the role of crosstalk between mast cells with sensory nerves as the contributors to neurogenic inflammation and pruritus in chronic skin inflammation.

The most widely accepted role of mast cells is the promotion of pain states. Theoharides et al. proposed that thalamic mast cells contribute to inflammation and pain in fibromyalgia by releasing multiple pro-inflammatory molecules which could either stimulate thalamic nociceptive neurons directly or activate microglia in the diencephalon. They also suggest stabilization of mast cells as the novel approach in treating this painful state.

Richards et al. demonstrate the discogenic back pain and mast cell/Proteinase Activated Receptor 2-mediated interactions in this disorder.

In this collection, the role of meningeal mast cells as the triggers of migraine pain and local inflammation, originally proposed by the co-Editor of this e-book, T. Theoharidis, is presented in several papers. The group of Jansen-Olesen, in the original study, explored the role of the newly emerged migraine mediator PACAP as the trigger of degranulation of meningeal mast cells via the atypical orphan MRGB3-receptor (Pedersen et al.).

The experimental study for Koroleva et al. shows, by using mice deficient in mast cells, that the putative migraine mediator extracellular ATP strongly activates nociceptive firing in meningeal trigeminal afferents via degranulation of resident mast cells and release of serotonin together with the direct excitatory action on the nerve terminals via purinergic receptors.

Irmak et al. review the role of meningeal mast cells in migraine pathology by further developing the concept of couplings between the sensory nerve fibers and meningeal mast cells. They highlight the bidirectional signaling between these key partners of the *neuro-immune synapse* and shared fate of them in generation of the persistent pain state specific for migraine.

The study by Nurkhametova et al. demonstrates the important contribution of P2X7 receptors to ATP-driven activation of meningeal mast cells, suggesting these purinergic mechanisms as potential triggers of neuroinflammation and pain sensitization in migraine.

Traina presents an interesting view on the contribution of intestinal microbiota to mood and behavioral disorders via microbiota–gut–brain axis.

Several papers analyze the role of mast cells in brain disorders and suggest the beneficial role of their stabilization as a promising therapeutic approach. Thus, the study by Dong et al. shows that the stabilization of brain mast cells alleviated neuroinflammation by inhibiting microglia activation.

The paper by Jones and Gupta provides the general overview of the field of mast cells in neuroinflammation and neurodegeneration with the focus on development and progression of four prominent neurodegenerative diseases: Alzheimer's Disease, Parkinson's Disease, Amyotrophic Lateral Sclerosis, and Huntington's Disease.

Laurino et al. shows that 3-iodothyroacetic acid degranulate hypothalamic mast cells to provide the antidepressant effect in the brain circuits via histamine signaling.

Arac et al. in the “Hypothesis and Theory” article, which is based on their recent original study, further develop the idea that meningeal mast cells are the key effectors in stroke pathology.

Landucci et al. present an interesting hypothesis that thyroid function may affect mast cell function, consequently, and they suggest that mast cell degranulation in the brain may impact thyroid function.

Several papers highlight the phenomenon and explore the underlaying mechanisms of mast cell-induced modulation of the blood-brain barrier permeability. Thus, Kempuraj et al. focuses on the pathogenic stress as the initial trigger in development of neurodegenerative diseases. Specially, they propose the important role of the stress associated corticotropin-releasing hormone (CRH) in activation of mast cells ultimately leading to neuroinflammation.

Tran et al. provide novel mechanistic insights into mast cell-induced blood-brain barrier damage in cerebrovascular diseases via endoplasmic reticulum stress in the endothelium.

In summary, this e-book presents the current state of knowledge about the involvement of mast cells in pain, itch, neuroinflammation, and associated neurological disorders. This collection identifies treatable targets for the development of novel pharmacologic agents and approaches for the treatment of pain and neuro-inflammatory disorders.

## Author's Note

The editorial is devoted to the memory of our colleague Stephen Skaper who passed away in 2018 soon after start of this project. Dr. Skaper's contribution to the field of mast cells will continue to guide the field, and his contribution to the editorial efforts of this issue are highly appreciated.

## Author Contributions

All authors listed have made a substantial, direct and intellectual contribution to the work, and approved it for publication.

### Conflict of Interest

The authors declare that the research was conducted in the absence of any commercial or financial relationships that could be construed as a potential conflict of interest.

